# Non-Uniform Distribution Pattern for Differentially Expressed Genes of Transgenic Rice Huahui 1 at Different Developmental Stages and Environments

**DOI:** 10.1371/journal.pone.0037078

**Published:** 2012-05-11

**Authors:** Zhi Liu, Jie Zhao, Yunhe Li, Wenwei Zhang, Guiliang Jian, Yufa Peng, Fangjun Qi

**Affiliations:** State Key Laboratory for Biology of Plant Diseases and Insect Pests, Institute of Plant Protection, Chinese Academy of Agricultural Sciences, Beijing, People's Republic of China; Kansas State University, United States of America

## Abstract

DNA microarray analysis is an effective method to detect unintended effects by detecting differentially expressed genes (DEG) in safety assessment of genetically modified (GM) crops. With the aim to reveal the distribution of DEG of GM crops under different conditions, we performed DNA microarray analysis using transgenic rice Huahui 1 (HH1) and its non-transgenic parent Minghui 63 (MH63) at different developmental stages and environmental conditions. Considerable DEG were selected in each group of HH1 under different conditions. For each group of HH1, the number of DEG was different; however, considerable common DEG were shared between different groups of HH1. These findings suggested that both DEG and common DEG were adequate for investigation of unintended effects. Furthermore, a number of significantly changed pathways were found in all groups of HH1, indicating genetic modification caused everlasting changes to plants. To our knowledge, our study for the first time provided the non-uniformly distributed pattern for DEG of GM crops at different developmental stages and environments. Our result also suggested that DEG selected in GM plants at specific developmental stage and environment could act as useful clues for further evaluation of unintended effects of GM plants.

## Introduction

With the development of transgenic technology, GM crops have increased the farm income dramatically during the past years [1]. However, there have been, and would continue to be, considerable public concerns for the commercialization of GM crops. Such concerns focus on whether random insertion of transgenes into host plant genomes would result in unpredicted changes in expression pattern of other intrinsic genes, leading to unintended effects on GM crops and their products [2]. It is generally agreed that unintended effects should be paid particular attention in the process of safety assessment of GM crops and their products, especially in regard to some long-term and potential food safety issues [3].

The use of profiling technologies, such as DNA microarray analysis, has been proved to be an effective way to detect differentially expressed genes (DEG) and investigate unintended effects in a number of transgenic plant systems. For example, Gregerson *et al.* compared the gene expression profiles of wild type wheat seeds and GM wheat seeds at three developmental phases using a 9K unigene cDNA microarray and found only slight differences in gene expression profiles [4]. Affymetrix *Arabidopsis* ATH1 GeneChip was used to search for transcriptome changes in *Arabidopsis* and the result turned out that the insertion and expression of the marker genes, *uidA* and *nptII*, did not induce changes to the expression profiles under optimal growth conditions and under physiological stress imposed by low temperatures [5]. Also, Affymetrix *Arabidopsis* ATH1 GeneChip was used to study the pleiotropic effects of the *bar* gene and glufosinate on the *Arabidopsis* transcriptome by detecting DEG [6]. Microarray analysis was performed on *Arabidopsis* plants overexpressing transcription factor ABF3, and no unintended effects were discovered [7].

However, the majority of researches investigating DEG and unintended effects of GM crops [4]–[14], were carried out using GM plants at specific developmental phases and/or particular environments. As a consequence, the results of such investigations might be invalid unless DEG and unintended effects of GM crops at specific developmental phases and/or environmental conditions could be representative for GM plants in all conditions.

The distribution of DEG under different conditions (developmental stages or environments), however, still remains unclear. Apparently, it is possible that the distribution pattern of DEG might vary under different conditions. Theoretically, there are three possible distribution patterns of DEG: (I) uniform distribution; the amount of DEG remain more or less constant regardless of developmental phases or environmental factors, (II) extreme distribution; the number of DEG differ dramatically in different conditions, with extremely huge amount of DEG in some conditions and a nominal sum of DEG in other conditions, (III) non-uniform distribution; DEG distribute randomly, with various considerable amount of DEG in different conditions. If DEG were uniformly distributed, then the DEG detected under any condition would be representative and valid for investigations of unintended effects. If DEG were non-uniformly distributed, then the number and distribution of DEG under different conditions might vary, but considerable DEG could still be detected, if there were, and unintended effects based on DEG were valid. If DEG were extremely distributed, however, the DEG were not representative and invalid for investigations of unintended effect, since extremely huge or nominal number of DEG might be detected under different conditions. So it is crucial to clarify the distribution of DEG before investigating unintended effects and assessing safety of GM plants.

Transgenic rice Huahui 1 (HH1) and its corresponding non-transgenic parent rice Minghui 63 (MH63) were used in this study. HH1 was an insect-resistant rice expressing *BT* fusion protein derived from *Cry1Ab* and *Cry1Ac*. HH1 was created by micro projectile bombardment with two plasmids, pFHBT1 and pGL2RC7, into the elite Chinese cytoplasmic male sterile restorer line, MH63. The plasmid pFHBT1 harbored a hybrid *Cry1Ab/Ac* gene regulated by the rice *actinI* gene promoter and the nopaline synthase (NOS) terminator; plasmid pGL2RC7 carried a Chitinase gene (*RC7*) and a selectable marker gene (*Hph*). The selectable marker gene *Hph* was further removed from the gene of interest by self-segregation [15], [16]. Field tests showed that production efficiency of HH1 was increased through resistance against yellow stem borers and leaf folders [17].

In this paper, with the aim to define the distribution pattern of DEG, we performed DNA microarray analysis on HH1 and MH63 at 4 different developmental stages and in 6 different environments (high temperature, low temperature and pathogen inoculations). DEG and significantly changed pathways of HH1 at different developmental stages and environments were analyzed. The results suggested that DEG were non-uniformly distributed in HH1 at different developmental stages and/or environments. Thus DEG detected by comparative transcriptome microarray analysis under certain conditions would be representative for DEG of GM plants under other conditions, and would act as valid clues for further investigation of unintended effects of GM plants.

## Results

### The insertion of *Cry1Ab/1Ac* did not cause differential expression of genes in insertional positions in HH1

To study whether or not there are DEG near the insertion site in HH1, we performed microarray analysis using HH1 and MH63 at different developmental stages (30-day, 60-day, 75-day and 90-day) and environmental conditions (temperature and pathogen stress) and investigated the expression level of genes located within 100 kb up-stream and 100 kb down-stream of the insertion site. According to the reported 3′- and 5′-franking sequences of the hybrid *Cry1Ab/1Ac* gene [15], BLAST analysis was performed. The result indicated that the hybrid *Cry1Ab/1Ac* gene was inserted into chromosome 10, between 5378530 and 5378531. There were 8 genes within 100 kb up- and down-stream of the insertion site. The expression levels of all these 8 genes were not obviously changed (with fold change between 0.5 and 2.0).

### Number of DEP on each rice chromosome in HH1 at different developmental stages and environments

In order to determine the global distribution pattern of DEG, we performed microarray analysis using HH1 and MH63 at different developmental stages and environmental conditions. Table 1 shows the distribution pattern of differentially expressed probe sets (DEP, with fold change ≥2.0 or ≤0.5) on each chromosome. In each case, the numbers of DEP on each chromosome were different: on chromosome 10, where *Cry1Ab/1Ac* was inserted, there were only a few DEP; on chromosome 12, there were also a small number of DEP; on chromosome 1, 5, 7, 9, etc, there were a large number of DEP. This result indicated that DEP were non-uniformly distributed on chromosome in HH1 at different developmental stages and environments.

**Table 1:** Number of DEP on each rice chromosome in HH1 at different developmental stages and environments.

	Chr1	Chr2	Chr3	Chr4	Chr5	Chr6	Chr7	Chr8	Chr9	Chr10	Chr11	Chr12
**30-day**	49	28	26	20	27	16	22	13	18	7	33	10
**HT**	13	9	6	5	10	3	17	4	15	4	34	4
**LT**	45	19	24	16	24	21	36	19	31	9	30	8
**60-day**	27	15	16	8	21	8	23	2	14	4	30	8
**75-day**	67	39	49	32	34	26	51	17	41	23	43	18
**90-day**	24	15	17	14	21	6	29	15	16	8	31	5
**JxoI**	19	17	19	14	14	8	27	10	23	8	37	7
**Pxo99**	54	34	51	36	34	19	37	19	34	10	45	13
**Rs105**	14	12	17	12	12	4	22	6	19	2	31	5
**Xv5**	13	14	15	7	18	6	27	4	22	3	34	3

### Identification of DEG responding to developmental stages and environmental conditions

**Figure 1:**
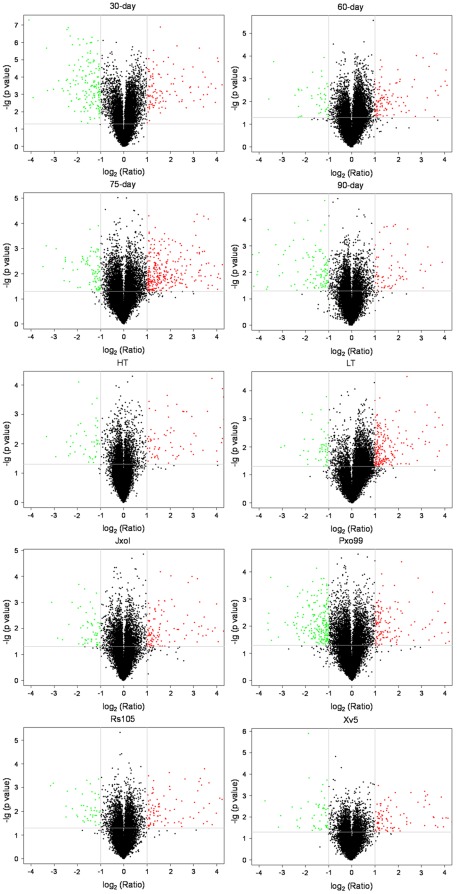
Volcano plots for differentially expressed genes in HH1. Each point represents a gene detected in microarray analysis. Red spots represent differentially expressed genes with fold change ≥2.0; green spots represent differentially expressed genes with fold change ≤0.5. The log_2_ (ratio) of expression (HH1/MH63) is shown on the X-axis and the –lg (p-value) is shown on the Y-axis. The vertical lines represent 2-fold change ratio and the horizontal line represents statistical-significance level where p = 0.05. 30-day, 60-day, 75-day, 90-day: HH1 and MH63 at developmental stage of 30-day, 60-day, 75-day, 90-day, respectively; HT: HH1 and MH63 treated with high-temperature at 45°C for 6 hours; LT: HH1 and MH63 treated with low-temperature at 12°C for 6 hours; JxoI, Pxo99: HH1 and MH63 inoculated with *X. oryzae* pv. *oryzae* JxoI and Pxo99 strain; Rs105: HH1 and MH63 inoculated with *X. oryzae* pv. *oryzicola* Rs105 strain; Xv5: HH1 and MH63 inoculated with non-host pathogen *X. compestris* pv. *vesicatoria* Xv5 strain.

To determine numbers of DEG at different developmental stages, we performed microarray analysis using HH1 and MH63 at the age of 30-day, 60-day, 75-day and 90-day, respectively. Considerable DEP were detected (Table 1, Table S1). Since some genes were represented by more than one probe, the corresponding numbers of DEG of HH1 at the four developmental stages were 261, 167, 422 and 195, respectively (Table 2). To explore numbers of DEG at different environmental conditions, we treated HH1 and MH63 with high-temperature (HT) and low-temperature (LT) at the age of 30-day and inoculated HH1 and MH63 with pathogens (JxoI, Pxo99, Rs105, Xv5) at the age of 75-day old, respectively, and performed microarray analysis. There were 116 and 271 DEG in HH1 treated with HT and LT, and 194, 372, 148 and 157 DEG in HH1 inoculated with JxoI, Pxo99, Rs105 and Xv5, respectively (Table 2). Furthermore, as shown in volcano plots (Figure 1), there were more down-regulated probes (with fold change ≤0.5) than up-regulated probes (with fold change ≥2.0) in the group of 30-day, and in all the other groups, there were more up-regulated probes than down-regulated probes, indicating that the number of up-regulated probes and down-regulated probes varied in HH1 at different conditions. These results indicated that considerable differentially expressed DEP and DEG could be detected in HH1 at different stages and environmental conditions.

**Table 2:** Numbers of DEP and DEG in HH1 compared with MH63 at different developmental stages and environments.

Treatments	Developmental stage				Temperature stress		Pathogen inoculation			
	30 d	60 d	75 d	90 d	HT	LT	JxoI	Pxo99	Rs105	Xv5
**DEP Number**	271	177	442	203	125	283	207	389	157	168
**DEG Number**	261	167	422	195	116	271	194	372	148	157

### Common DEP among HH1 at different developmental stages and environmental conditions

**Figure 2:**
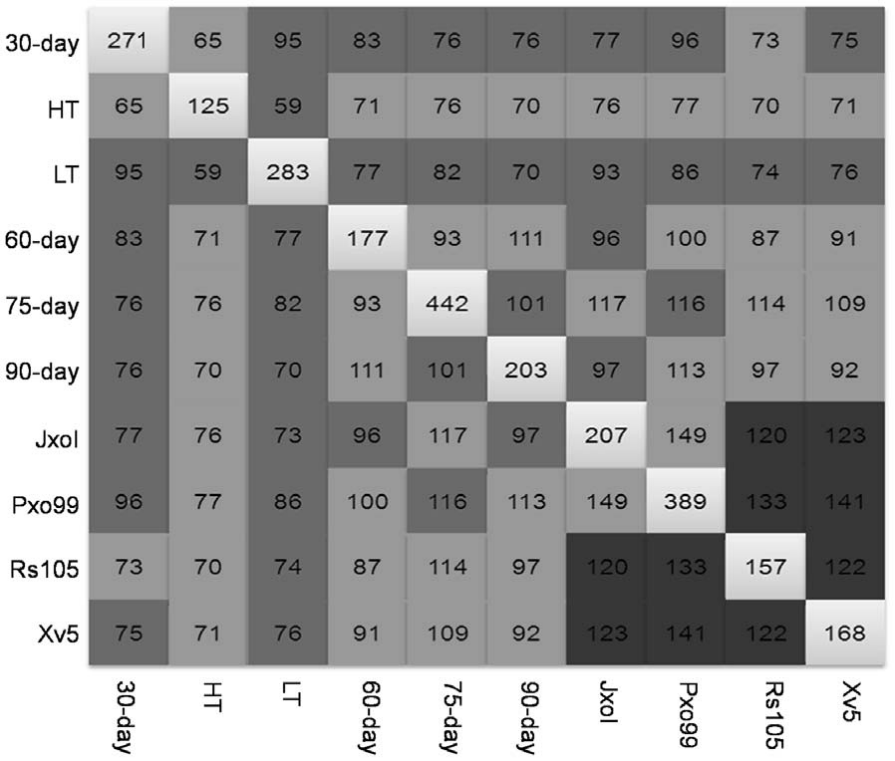
Distribution patterns of common DEP of HH1 in pairwise comparison. Pairwise comparisons were carried out between groups of DEP of HH1 at different developmental stages and environments. Ratio of number of common DEP to number of the smaller group of DEP in the pairwise comparison was calculated and represented by different colors: ▪- 25∼50%, ▪- 50∼75%, ▪- 75∼100%. ▪represents numbers of DEP in HH1. 30-day, 60-day, 75-day, 90-day: HH1 and MH63 at developmental stage of 30-day, 60-day, 75-day, 90-day, respectively; HT: HH1 and MH63 treated with high-temperature at 45°C for 6 hours; LT: HH1 and MH63 treated with low-temperature at 12°C for 6 hours; JxoI, Pxo99: HH1 and MH63 inoculated with *X. oryzae* pv. *oryzae* JxoI and Pxo99 strain; Rs105: HH1 and MH63 inoculated with *X. oryzae* pv. *oryzicola* Rs105 strain; Xv5: HH1 and MH63 inoculated with non-host pathogen *X. compestris* pv. *vesicatoria* Xv5 strain.

In order to clarify whether there were common DEP among HH1 at different developmental stages and environmental conditions, we performed pairwise comparisons between each group of DEP. It turned out that there were considerable common DEP between each group of HH1 (Figure 2, Table S2). The ratio of number of common DEP to number of the smaller group of DEP in the comparison was calculated and represented by different boxes (Figure 2). The numbers of common DEP were not proportional to the numbers of DEP in each group of HH1. Furthermore, numbers of common DEP ranked from 59 (HT and LT) to 149 (Pxo99 and JxoI). These results suggested that common DEP between each group of HH1 were neither uniformly distributed nor extremely distributed; instead, they were non-uniformly distributed and the amount of common DEP in each group of HH1 was adequate for investigating unintended effects.

### Significantly changed pathways among HH1 at different developmental stages and environmental conditions

To further explore influences of DEG on HH1, we analyzed changes in pathways of HH1 using Plant MetGenMAP system. A number of significantly changed pathways were selected. Among these significantly changed pathways, 16 pathways were found in all groups of HH1, 8 pathways were found in the majority of groups of HH1 (Table 3), and the other significantly changed pathways dispersed in each group of HH1 (Table S3). This finding indicated that a certain number of common significantly changed pathways were shared among HH1 under different conditions. These changes, with the mere differences at expression level, were everlasting existed in HH1, suggesting that they were probably caused by genetic modification rather than differences in developmental stages and/or environments.

**Table 3:** Common significantly changed pathways of HH1 at different developmental stages and environments.

Pathway name	*p* value									
	30-day	HT	LT	60-day	75-day	90-day	JxoI	Pxo99	Rs105	Xv5
jasmonic acid biosynthesis	√	√	√	√	√	√	√	√	√	√
enterobactin biosynthesis	√	√	√	√	√	√	√	√	√	√
sucrose degradation to ethanol and lactate (anaerobic)	√	√	√	√	√	√	√	√	√	√
oxidative ethanol degradation I	√	√	√	√	√	√	√	√	√	√
phenylalanine degradation III	√	√	√	√	√	√	√	√	√	√
methionine degradation III	√	√	√	√	√	√	√	√	√	√
ethanol fermentation to acetate	√	√	√	√	√	√	√	√	√	√
valine degradation II	√	√	√	√	√	√	√	√	√	√
tetrapyrrole biosynthesis I	√	√	√	√	√	√	√	√	√	√
leucine degradation III	√	√	√	√	√	√	√	√	√	√
mixed acid fermentation	√	√	√	√	√	√	√	√	√	√
isoleucine degradation II	√	√	√	√	√	√	√	√	√	√
cytokinins 7-N-glucoside biosynthesis	√	√	√	√	√	√	√	√	√	√
cytokinins 9-N-glucoside biosynthesis	√	√	√	√	√	√	√	√	√	√
betanidin degradation	√	√	√	√	√	√	√	√	√	√
cytokinins-O-glucoside biosynthesis	√	√	√	√	√	√	√	√	√	√
aerobic respiration – electron donor II	√	√	√	√	X	√	√	√	√	√
photorespiration	√	X	√	√	√	√	√	√	√	√
NAD salvage pathway II	√	√	√	√	√	X	√	√	X	√
brassinosteroid biosynthesis II	√	√	√	√	√	√	X	√	X	√
medicarpin biosynthesis	√	X	X	√	√	√	X	√	X	√
maackiain biosynthesis	√	X	X	√	√	√	X	√	X	√
cellulose biosynthesis	X	√	X	X	√	√	√	√	√	X
starch degradation	X	X	X	X	√	√	√	√	√	√

30-day, 60-day, 75-day, 90-day: HH1 and MH63 at developmental stage of 30-day, 60-day, 75-day, 90-day, respectively; HT: HH1 and MH63 treated with high-temperature at 45°C for 6 hours; LT: HH1 and MH63 treated with low-temperature at 12°C for 6 hours; JxoI, Pxo99: HH1 and MH63 inoculated with *X. oryzae* pv. *oryzae* JxoI and Pxo99 strain; Rs105: HH1 and MH63 inoculated with *X. oryzae* pv. *oryzicola* Rs105 strain; Xv5: HH1 and MH63 inoculated with non-host pathogen *X. compestris* pv. *vesicatoria* Xv5 strain. √: detected; X: not found (For detailed information, please refer to Table S3).

## Discussion

GM plants, first planted in 1996, have occupied 148 million hectares cropland in 2010, nearly 10% of all 1.5 billion hectares cropland in the world [18]. Compared to traditional breeding approaches, transgenic approach is direct and breaks the reproductive isolation, with which scientists can transfer any gene-of-interest from any species into chosen crops [19]. Despite the many benefits of the GM crops [18], people are concerned about safety of GM crops and products derived from them. Since random insertion of exogenous specific DNA sequences into plant genome may cause disruption, modification or silencing of active genes and/or activation of silenced genes, resulting in unintended effects [19].

Detecting unintended effects is an important task in safety assessment of GM crops. Traditional methods to detect unintended effects, such as comparing agronomic characters, evaluating environmental adaptability, and analyzing the chemical compositions between GM and non-GM plants [20], are considered as targeted approaches; the limitations of these methods are obvious, especially in the aspects of time and economic consuming and lack of objectivity and impartiality [21], [22]. With technical breakthroughs in recent years, DNA microarray has emerged as an indispensable methodology for large-scale and high-throughput analysis of genes in the crops. DNA microarray is a non-targeted approach, and has been proved to be an effective and comprehensive method to detect DEG and investigate unintended effects in GM crops [4]–[7]. Most of these studies focus on DEG and unintended effects of GM plants at specific developmental stage and/or particular environmental condition and neglect the fact that certain factors, such as developmental stages and environmental factors, may influence distribution pattern of DEG, which further may influence the occurrences of unintended effects. Without detailed evidence on the distribution pattern of DEG of GM plants at different developmental stages and/or environments, it might be questionable to investigate unintended effects using GM plants at specific developmental stages and/or particular environments.

Thus, it is necessary to understand the distribution pattern of DEG in GM plants at different developmental stages and/or environments before investigating unintended effects. As discussed above, there are three possible distribution patterns of DEG: (I) uniform distribution, (II) extreme distribution and (III) non-uniform distribution. DEG detected from microarray analysis were valid for further predicting unintended effects if they were uniformly distributed or non-uniformly distributed; if DEG were extremely distributed, however, DEG detected from microarray analysis were invalid for assessment of unintended effects, since DEG were not representative.

In this study, with the purpose of revealing the distribution pattern of DEG in GM plants, we performed microarray analysis with groups of HH1 and MH63 at different developmental stages and environments. No DEG were found near the insertion site (100 kb up- and down-stream of the insertion site), suggesting the transgene event did not cause changes on expression level of intrinsic genes near the insertion site. In each case, the numbers of DEP, detected in microarray analysis, on each chromosome were difference, indicating DEG on each chromosome was non-uniformly distributed. Considerable DEG were found in each group of HH1, and the numbers of DEG varied with changes in developmental stages and/or environments (Table 2). We found that distribution pattern of DEG in HH1 was closest to the non-uniform distribution pattern discussed above, so we conclude that DEG in HH1 was non-uniformly distributed. In addition, we investigate the relationship between DEP and growing conditions (developmental stages and/or environmental conditions in which GM plants are growing) of GM plants. If the number of DEP detected in different cases is relevant to growing conditions, then the numbers of DEP detected in HH1 at growing conditions would be about the same and the numbers of DEP detected in HH1 at different growing conditions would be significantly different. So we carried out pairwise comparisons using DEP detected in each case. The growing conditions of HH1 in our study could be classified into three types: different developmental stages (30-day, 60-day, 75-day and 90-day), temperature stress (HT and LT) and pathogen stress (JxoI, Pxo99, Rs105 and Xv5). As shown in Figure 2, the numbers of DEP in all the three types of growing conditions were around 200 (except for the case of 75-day and the case of Pxo99). The numbers of DEP in all the three types of growing conditions were about the same and no significant differences in the numbers of DEP were found between HH1 at different types of growing conditions, indicating that DEP had no relevance to growing conditions of HH1. This irrelevance is especially obvious in the case of pathogen stress. JxoI, Pxo99 and Rs105 are pathogenic pathogens that cause diseases on rice plants, and Xv5 is a non-host pathogen that does not cause any diseases on rice plants; the stresses caused by pathogenic pathogens and non-host pathogen are totally different. So the growing conditions in these four cases could be subdivided into two types: pathogenic stress and non-pathogenic stress. The numbers of DEP in HH1 under pathogenic stresses and non-pathogenic stress, however, remained about the same. So we concluded that the number of DEP in HH1 was not relevant to growing conditions. For the same reason, we got the conclusion that the number of common DEP was also not relevant to growing conditions of HH1. Moreover, Figure 2 showed that the number of common DEP in each case was large enough to be valid for assessment of unintended effects and were not proportional to the numbers of DEP. Based on these findings, we concluded that both DEG and common DEG were non-uniformly distributed, and the numbers of DEG and common DEG detected in HH1 had no relevance to growing conditions and the numbers of DEG and common DEG detected in HH1 at specific conditions were large enough to be representative and valid for investigating unintended effects.

Furthermore, we analyzed changes in expression level of pathways of HH1, and selected a number of significantly changed pathways. Among these significantly changed pathways, 16 pathways were found in all groups of HH1 (Table 3), 8 pathways were found in the majority of groups of HH1 (Table 3). These common DEG and significantly changed pathways in HH1 were probably to be caused by insertion of exogenous DNA fragment and had nothing to do with other factors, such as developmental stages and/or environmental factors. Among these common significantly changed pathways, jasmonic acid biosynthesis [23], medicarpin biosynthesis [24], and maackiain biosynthesis [24] were associated with response to biotic and abiotic stress. These changes were possibly intended effects of HH1, since HH1 were genetically modified to be resistant to pest insects. However, five common significantly changed pathways (Table 3), phenylalanine degradation III, methionine degradation III, valine degradation II, leucine degradation III, isoleucine degradation II, were associated with amino acid degradation. So it was necessary to carry out further research to determine whether these changes were intended effects or unintended effects.

Our finding provided evidences on the non-uniform distribution pattern of DEG in GM plants. So we could use DEG, especially common DEG and common significantly changed pathways, as a clue to investigate unintended effects of GM plants in future safety assessment of GM plants. However, DEG do not always mean unintended effects, since some DEG are directly associated with the transgenes introduced or with the desired new characteristics of GM plants. Further works should focus on distinguishing whether these DEG are associated with intended effects or unintended effects.

## Materials and Methods

### Plant Materials

Transgenic rice line Huahui 1 (HH1) and its corresponding non-transgenic line Minghui 63 (MH63) were used for microarray analysis. HH1 was genetically engineered to be insect-resistant through expressing fused insect-resistant gene of *Cry1Ac/Cry1Ab* by Huazhong Agricultural University, and obtained the first security certificate for genetically modified rice in China from Hubei Province in 2009 [25].

### Rice sample preparation

Rice seeds were surface-disinfected and then soaked in distilled sterile water for germination at 28°C for 2 days. Rice seedlings were grown in pots fertilized with half-strength of basal macro- and micro-salt nutrition components of Murashige and Skoog medium [26] in controlled climate chambers at 16-h-light (30°C)/8-h-dark (26°C) cycle. At the age of 30-day, 60-day, 75-day and 90-day old, seedling samples were collected, frozen in liquid nitrogen and kept at −80°C. Seedlings, at the age of 30-day old, were treated with high-temperature (45°C) and low-temperature (12°C) respectively at climate chambers, and samples were collected 6 hours after treatment. Seedlings were inoculated respectively with compatibility pathogen *Xanthomonas* oryzae pv. *oryzae* (rice leaf blight disease pathogen) JxoI and Pxo99 strains, *Xanthomonas* oryzae pv. *oryzicola* (rice leaf streak disease pathogen) Rs105 strain and rice non-host pathogen of *Xanthomonas compestris* pv. *vesicatoria* Xv5 strain at the age of 75-day old according to leaf rubbing inoculation method [27], and samples were collected 2 days after inoculation. All samples were kept at −80°C until RNA extraction. Each treatment was performed with three replicates, and more than 20 whole seedlings were collected for each sample.

### RNA extraction and microarray hybridization

Total RNA was extracted from rice samples according to the manufacturer's instructions of TRIzol reagent (Invitrogen Life Technologies, Carlsbad, CA, USA). The integrity of extracted RNA was checked and then sent to CapitalBio Corporation (an Affymetrix platform service facility at Beijing) for further quality and quantity examination and microarray hybridization. 1 µg of RNA samples was used for hybridization with Affymetrix GeneChip® Rice Genome Arrays according to the manufacturer's instructions. The array was designed mainly based on the annotation of TIGR version 2.0 and contained 55, 515 probe sets to query 48,564 transcripts of rice japonica subspecies and 1,260 transcripts of rice *indica* subspecies. Microarray hybridization was performed at 45°C with rotation lasting for 16 h using an Affymetrix GeneChip Hybridization Oven 640. Following hybridization, the arrays were washed and stained at Affymetrix GeneChip Fluidics Station 450 and then scanned with Affymetrix GeneChip® Scanner 3000 7G.

### Data analysis

The scanned images were analyzed with Affymetrix GeneChip® Command Console™ (AGCC) software. The expression flags (indicators of expressed genes) were determined using the Affymetrix® Expression Console™ software application MAS 5.0 algorithm as “present”, “marginal” and “absent” calls. Then normalization and expression analysis were performed with .CEL files and .mas5.CHP files by DNA-chip analyzer (dChip). All these data were deposited in NCBI GEO database with accession number GES33204. DEP and their corresponding DEG in HH1 were selected using the Significance Analysis of Microarrays (SAM version 3.02) software by two class unpaired method with q value ≤5% and fold change ≥2.0 or ≤0.5 when compared with control samples (MH63).

### Analysis of significantly changed pathways

Significantly changed pathways of HH1, in comparison with MH63, were analyzed by the Plant MetGenMAP system [28]. All changed pathways were selected by the raw *p* value with the threshold 0.05. Significantly changed pathways were selected by the FDR (False Discovery Rate) corrected *p* value with threshold 0.05 [29].

## Supporting Information

Table S1 **Differentially expressed probe sets in HH1.** Differentially expressed probe sets (DEP) in HH1 were selected using the Significance Analysis of Microarrays (SAM version 3.02) software by two class unpaired method with q value ≤5% and fold change ≥2.0 or ≤0.5 when compared with MH63. DEP with fold change ≥2.0 were represented in green color, and DEP with fold change ≤0.5 were represented in red color. 30-day, 60-day, 75-day, 90-day: HH1 and MH63 at developmental stage of 30-day, 60-day, 75-day, 90-day, respectively; HT: HH1 and MH63 treated with high-temperature at 45°C for 6 hours; LT: HH1 and MH63 treated with low-temperature at 12°C for 6 hours; JxoI, Pxo99: HH1 and MH63 inoculated with X. oryzae pv. oryzae JxoI and Pxo99 strain; Rs105: HH1 and MH63 inoculated with X. oryzae pv. oryzicola Rs105 strain; Xv5: HH1 and MH63 inoculated with non-host pathogen X. compestris pv. vesicatoria Xv5 strain.
(XLS)

Table S2 **Commom Differentially expressed probe sets (DEP) among HH1 at different developmental stages and enviromental conditions.** Differentially expressed probe sets (DEP) in HH1 were selected using the Significance Analysis of Microarrays (SAM version 3.02). Commen DEP Counted reprecented the number of DEP among HH1 at different developmental stages and enviromental conditions. “1” represent the absense of DEP. 30-day, 60-day, 75-day, 90-day: HH1 and MH63 at developmental stage of 30-day, 60-day, 75-day, 90-day, respectively; HT: HH1 and MH63 treated with high-temperature at 45°C for 6 hours; LT: HH1 and MH63 treated with low-temperature at 12°C for 6 hours; JxoI, Pxo99: HH1 and MH63 inoculated with X. oryzae pv. oryzae JxoI and Pxo99 strain; Rs105: HH1 and MH63 inoculated with X. oryzae pv. oryzicola Rs105 strain; Xv5: HH1 and MH63 inoculated with non-host pathogen X. compestris pv. vesicatoria Xv5 strain.
(XLS)

Table S3 **Significantly changed pathways in HH1 at different developmental stages and environmental conditions.** 30-day, 60-day, 75-day, 90-day: HH1 and MH63 at developmental stage of 30-day, 60-day, 75-day, 90-day, respectively; HT: HH1 and MH63 treated with high-temperature at 45°C for 6 hours; LT: HH1 and MH63 treated with low-temperature at 12°C for 6 hours; JxoI, Pxo99: HH1 and MH63 inoculated with X. oryzae pv. oryzae JxoI and Pxo99 strain; Rs105: HH1 and MH63 inoculated with X. oryzae pv. oryzicola Rs105 strain; Xv5: HH1 and MH63 inoculated with non-host pathogen X. compestris pv. vesicatoria Xv5 strain. ××: not found.
(XLS)
